# Pulmonary involvement in Anti‐Neutrophil Cytoplasmic Antibody Associated Vasculitis: A single centre case series

**DOI:** 10.1002/rcr2.1058

**Published:** 2022-10-21

**Authors:** Peter T. Bell, Robert Sheehy, Luke Droney, Kerri Prain, Richard Wong, Gregory J. Keir

**Affiliations:** ^1^ Department of Respiratory and Sleep Medicine Princess Alexandra Hospital Brisbane Queensland Australia; ^2^ Faculty of Medicine The University of Queensland Brisbane Queensland Australia; ^3^ Department of Immunology Princess Alexandra Hospital Brisbane Queensland Australia; ^4^ Division of Immunology Pathology Queensland Brisbane Queensland Australia

**Keywords:** antineutrophil cytoplasmic antibody, cyclophosphamide, rituximab, vasculitis

## Abstract

Anti‐Neutrophil Cytoplasmic Antibody associated Vasculitides (AAV) comprise a rare group of disorders in which respiratory tract involvement is variable and often severe. The rarity and heterogeneity of AAV makes this a challenging condition to diagnose and manage. In this single‐centre case series of 44 patients with AAV‐associated respiratory disease, we provide an overview of disease manifestations, management aspects and treatment outcomes. Data from this case series highlight the real‐world diagnostic and therapeutic challenges of the AAV respiratory disease spectrum; including uncertainties in the management of fibrosing interstitial lung disease, tracheobronchial stenosis and diffuse alveolar haemorrhage.

## Graphical abstract

Here, we describe the spectrum of disease manifestations and treatment outcomes in a large single‐centre case series of patients with Anti‐Neutrophil Cytoplasmic Antibody vasculitis ‐associated respiratory disease. This series highlights the diagnostic and treatment challenges encountered by physicians involved in the care of these patients, including the management of diffuse alveolar haemorrhage, relapsing tracheobronchial disease, and fibrosing interstitial lung disease.

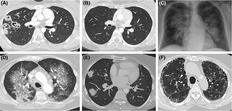

## INTRODUCTION

Anti‐Neutrophil Cytoplasmic Antibody (ANCA) associated Vasculitides (AAV) comprise a heterogeneous group of disorders characterized by small‐vessel necrotising vasculitis.^1^ Patients with AAV present with a spectrum of multi‐system manifestations, ranging from organ‐ or life‐threatening through to more indolent disease presentations.^1^ Respiratory tract involvement is highly variable, difficult to treat, and often associated with substantial morbidity.^2^

A recent Editorial published in the *Internal Medicine Journal* ^3^ outlines key challenges in the care of patients with vasculitis in Australia and New Zealand. One such challenge is the paucity of data describing local epidemiology and treatment outcomes,^3^ with even fewer detailed accounts of pulmonary manifestations of AAV. Here, we characterize the spectrum of disease manifestations and treatment outcomes in a large single‐centre case series of patients with AAV‐associated respiratory disease. This case series highlights diagnostic and treatment challenges encountered by physicians involved in the care of these patients, including the management of diffuse alveolar haemorrhage (DAH), relapsing tracheobronchial disease, and fibrosing interstitial lung disease (ILD).

## METHODS

This study was performed in compliance with the Declaration of Helsinki and according to approval granted by the local HREC (LNR/2021/QMS/74115). Respiratory disease was defined as either upper respiratory tract involvement (including middle ear and sinonasal disease), or lower respiratory tract involvement (including large airway, parenchymal or pleural disease). Serological testing was conducted in a single laboratory in accordance with the manufacturers recommendations. Patients with eosinophilic granulomatosis with polyangiitis (EGPA) were excluded due to pathobiological and treatment differences compared to other AAV phenotypes. Consecutive patients with AAV‐associated respiratory disease seen by the multidisciplinary vasculitis clinic at the Princess Alexandria Hospital between January 2015 and August 2021 were included in this case series. The Princess Alexandria Hospital is a university teaching hospital and referral centre for vasculitis. The multidisciplinary clinic comprises respiratory physicians and immunologists with expertise in the diagnosis and management of small vessel vasculidities, with collaborative input from nephrologists and otorhinolaryngologists in managing extra‐pulmonary disease manifestations. Data analyses were performed using non‐parametric statistics.

### Case series

Forty‐four patients with a diagnosis of AAV‐associated respiratory disease were identified over the 5½ year study period, including 34 patients with granulomatosis with polyangiitis (GPA) (77.3%), seven with microscopic polyangiitis (MPA) (15.9%), and three with unclassifiable AAV (6.8%). Median age at diagnosis was 55 years (range 12–84), with a female preponderance (*n* = 27; 61.4%). Median follow‐up duration at time of data censoring was 7.5 years. Demographic, clinical and serologic data are summarized in Table 1.

**TABLE 1:** Clinical and demographic data for patients with AAV

	GPA	MPA	Unclassifiable	Total	*p*‐Value^a^
Patients, *n* (%)	34 (77.3)	7 (15.9)	3 (6.8)	44 (100)	–
Gender (F/M)	21/13	4/3	2/1	27/17	1.0
Age at diagnosis, *median (range)*	49 (18–77)	64 (48–84)	65 (59–69)	55 (18–84)	0.04
ANCA Serology (PR3/MPO/ANV)	27/11/6	0/7/0	0/2/1	27/11/6	–
Respiratory tract involvement					
LRT involvement (%)	82.4	71.4	100	84.1	1.0
URT involvement (%)	67.6	14.3	66.7	59.1	0.01
Extra‐respiratory tract involvement (%)	50.0	85.7	100	59.1	0.11
Pulmonary nodule/mass (%)	64.7	14.3	33.3	54.5	0.03
Diffuse alveolar haemorrhage (%)	8.8	42.9	33.3	15.9	0.05
Fibrosing ILD (%)	0	42.9	33.3	8.9	<0.01
Sinonasal disease (%)	67.6	14.3	66.7	59.1	0.01
Otitis media (%)	17.6	0	33.3	15.9	0.57
Sensorineural hearing loss (%)	14.7	0	33.3	13.6	0.57
Ocular Involvement (%)	8.8	0	0	6.8	1.0
Tracheobronchial disease (%)	17.6	0	0	13.6	0.57
Pleural effusion (%)	5.9	0	0	4.5	1.0
Pauci‐immune necrotizing GN (%)	14.7	71.4	66.7	27.3	<0.01
CKD^b^ (%)	5.9	57.1	66.7	18.1	<0.01
Cutaneous disease (%)	11.8	0	33.3	11.4	1.0
Peripheral neuropathy (%)	2.9	0	66.7	6.8	1.0
Relapse^c^ (%)	55.9	14.3	33.3	47.7	0.09

Abbreviations: CKD, Chronic Kidney Disease; ILD, interstitial lung disease; LRTI, lower respiratory tract; MPO, myeloperoxidaseANCA autoantibody; PR3, proteinase‐3 ANCA autoantibody; URTI, upper respiratory tract.

^a^ Statistical tests performed comparing GPA and MPA phenotypic groups.

^b^ Significant chronic kidney disease defined as CKD Stages III–V as a result of AAV‐Glomerulonephritis.

^c^ Relapse defined as clinical relapse requiring re‐induction immunosuppresion.

Lower respiratory tract involvement was present in 37 patients (84.1%); with pulmonary nodules/consolidation the most frequent manifestation (*n =* 24; 54.5%). Nodules were more common in GPA (64.7%) compared to MPA (14.3%; *p =* 0.03) and were often the index disease manifestation in GPA. DAH occurred in seven patients (15.9%) at time of diagnosis (*n* = 4 MPO‐ANCA+; *n* = 3, PR3‐ANCA+). Patients with DAH were predominantly female (*n =* 4, 57.1%), significantly older than the non‐DAH cohort (median age, 65 years [range 48–84] vs 48 years [range 12–77]; *p =* 0.02), and the majority (*n =* 6; 85.7%) had concomitant renal vasculitis. Three patients received total plasma exchange (TPE), in addition to cyclophosphamide‐containing induction therapy. No patient had recurrent DAH after treatment was commenced and all remain alive after a median duration follow‐up of 6.1 years. In this series, index presentations with AAV disease were highly variable across individual patients and usually involved unique combinations of protean symptoms, reflecting the multisystem nature of this disease. Symptoms at index presentation varied as a function of disease manifestation(s); however, most frequently included dyspnoea, cough and sinus discomfort often with associated purulent/blood‐stained discharge. Table 1 provides additional information on sites of disease.

**FIGURE 1:**
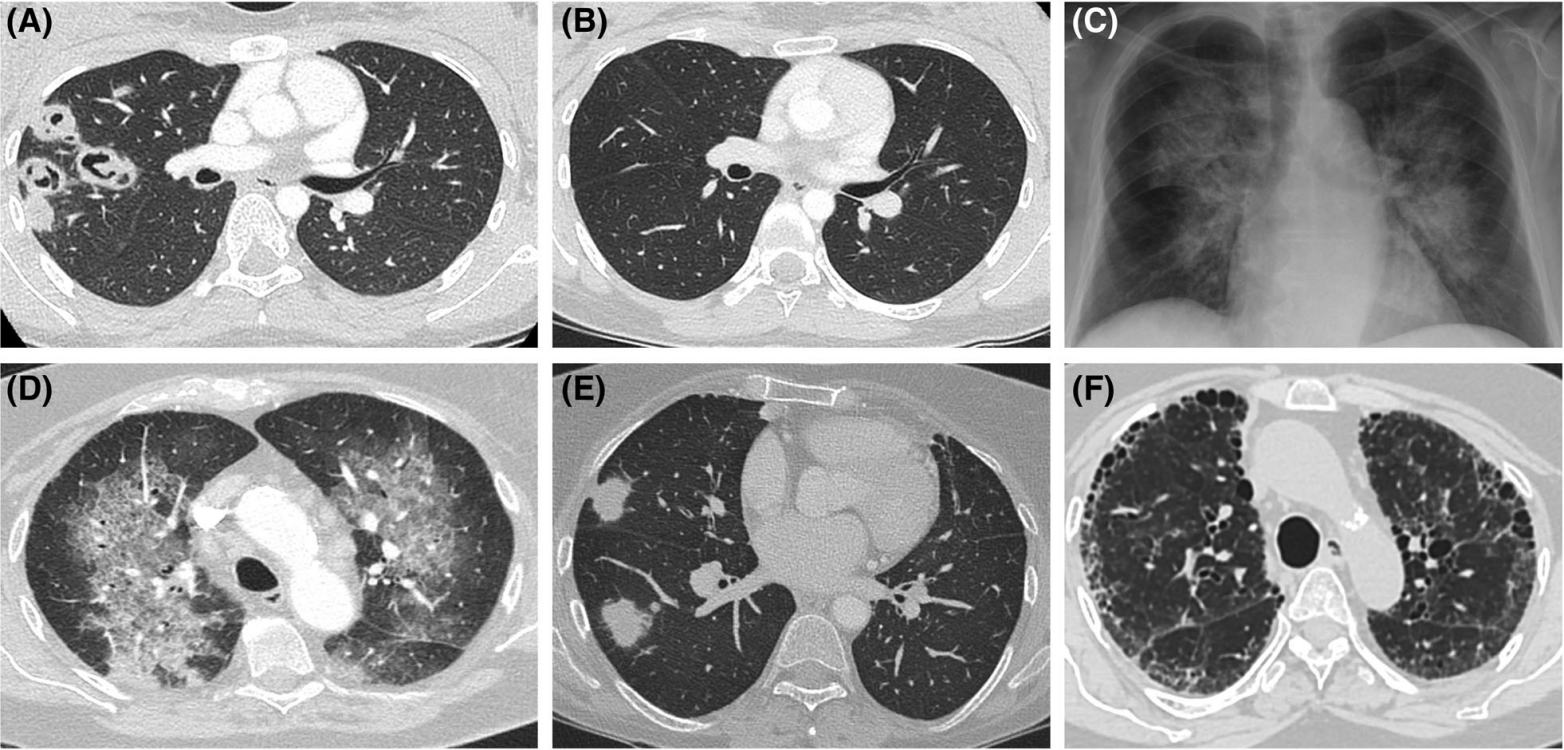
Heterogeneity of pulmonary involvement in ANCA associated vasculitis. (A) CT scan (axial plane) of an 18‐year‐old female presenting with multiple right upper lobe cavitating nodular lesions associated with pleuritic chest pain and concomitant rapidly progressive sensorineural hearing loss. She was diagnosed with PR3‐ANCA+ GPA and underwent induction with rituximab. (B) Following induction, there was complete radiologic resolution at 5 months and ANCA seroconversion at 8 months. (C, D) Posteroanterior (PA) chest radiograph and CT scan (axial plane) of an 84‐year‐old female presenting with dyspnoea and normocytic anaemia (haemoglobin 80 g/L on presentation). Radiology demonstrates typical appearance of diffuse alveolar haemorrhage with central peribronchovascular ground glass attenuation with subpleural sparing. (E) Multifocal nodular/mass lesions in a 62‐year‐old female with PR3 ANCA+ GPA. CT‐guided percutaneous biopsy of the right lobe lesion revealed chronic inflammation and fibrosis, without definite histological features of GPA. (F) 75‐year‐old female with a history of treated MPO ANCA+ MPA with isolated renal involvement at diagnosis, subsequently went on to develop advanced pulmonary fibrosis with honeycombing (usual interstitial pneumonia) over a decade later.

Large airway involvement with tracheobronchial stenosis occurred in six patients, all of whom had GPA (*n* = 4 PR3‐ANCA+; *n* = 2 ANCA‐negative AAV [ANV]). These patients were exclusively female and were significantly younger than the remaining cohort (median age, 41.5 years [range 12–48] vs 59 years [range 18–84]; *p =* 0.01). In addition to immunosuppression, all patients with tracheobronchial disease required repeated endobronchial intervention(s) due to recurrent airway stenosis. Four patients (median age, 68.5 years [range 61–74]) had fibrosing interstitial lung disease (ILD), all with a radiological pattern of usual interstitial pneumonia (UIP) (Figure 1). Of these, three had MPO‐ANCA+ MPA, and one patient had ANCA‐negative serology with GPA/MPA phenotypic overlap. In three patients, systemic manifestations of vasculitis (including renal involvement, skin rash and digital ischaemia) preceded ILD diagnosis. The remaining patient had idiopathic pulmonary fibrosis (IPF) and concomitant high titre MPO‐ANCA+ serology at time of diagnosis, with evolution into overt systemic vasculitis 16 months later.

Ten patients (22.3%) received rituximab‐based induction regimens, 33 received intravenous cyclophosphamide induction, and one patient did not receive immunosuppression (due to limited, non‐progressive pulmonary disease and advanced age). Corticosteroids, either intravenous methylprednisolone or high dose oral prednisolone, was administered in all induction regimens. All patients achieved clinical remission following induction therapy, with ANCA seroconversion in 26 patients (77.8%). Since January 2018 all patients have received rituximab‐based induction therapy upon diagnosis. Following induction, azathioprine or mycophenolate were the most frequently used agents for maintenance of remission. See Table 2 for detailed treatment data.

**TABLE 2:** Treatment paradigms for patients with AAV

	Rituximab induction^a^	Cyclophosphamide induction	*p*‐Value
Patients, *n* (%)	10 (22.7)	33 (75.0)	–
Gender (F/M)	9/2	18/15	1.0
Age at diagnosis, *median (range)*	53 (18–84)	54 (12–74)	0.65
Phenotype (GPA/ MPA/ Unclassifiable)	9/1/0	24/6/3	0.70
Seroconversion (%)^b^	88.9	74.1	0.65
Plasmapheresis (%)	0	9.1	1.0
Secondary hypogammaglobulinemia (%)^c^	50.0	28.1	0.26
Relapse (%)^d^	10.0	57.1	–

^a^ One patient with pulmonary‐renal syndrome received combination of both rituximab and intravenous cyclophosphamide induction immunosuppression.

^b^ ANCA seroconversion rate reported in those patients with ANCA‐positive AAV.

^c^ One patient was excluded from this analysis due to primary hypogammaglobulinemia in the context of Combined Variable Immunodeficiency (CVID).

^d^ Relapse defined as clinical relapse requiring reinduction therapy.

AAV relapse, defined as recurrence of pulmonary or extra‐pulmonary disease requiring re‐induction therapy, occurred in 21 patients (47.7%). A trend towards greater risk of relapse was seen with GPA (55.9%) compared with MPA (14.3%; *p =* 0.09). To date, only one patient (*n =* 1/10) experienced disease relapse requiring reinduction therapy after initial rituximab‐based induction therapy (median follow up time = 34.5 months); whereas 57.1% (*n* = 20/35) of patients in the cyclophosphamide group have had disease relapse during follow‐up (median follow‐up time = 111 months). In all patients with relapse, rituximab was used as reinduction therapy, resulting in clinical remission and complete peripheral blood CD19+ B‐cell depletion in all patients (CD19+ absolute cell count <0.01 × 10^9^).

## DISCUSSION

The rarity and heterogeneity of AAV makes this a difficult condition to diagnose and manage. This single‐centre case series highlights the real‐world clinical challenges encountered by physicians involved in the care of patients with AAV respiratory disease; including uncertainties in the management of fibrosing ILD, tracheobronchial stenosis and DAH.

Index manifestations of AAV‐respiratory disease can masquerade as neoplasia and pulmonary infection, leading to diagnostic uncertainty and delay. Granulomatous inflammation of pulmonary parenchyma can result in nodule/mass formation (either solitary or multiple) that frequently cavitate due central necrosis.^2^ In multiple instances in this case series, the differential diagnosis of AAV was only considered only after obtaining results of invasive tissue sampling of pulmonary nodules. In other cases, the possibility of infection or malignancy arose subsequent to immunosuppressive treatment for known AAV and required invasive diagnostic sampling.

There is an increasingly recognized association between AAV (particularly MPA) and fibrotic ILD.^4^ with some experts proposing that ILD might even represent a form of pulmonary‐limited MPA.^5^ ILD may occur antecedent, synchronous or subsequent to the onset of overt systemic vasculitis,^6^ and most patients with MPA‐ILD have a radiologic pattern of UIP.^7^ In our series, we have seen patients develop progressive fibrosing ILD several years after diagnosis with MPA. The contrary scenario was also observed, in which a patient with fibrosing ILD and positive MPO‐ANCA antibody serology at diagnosis subsequently evolved into overt systemic vasculitis. Currently, the ATS/ERS/JRS/ALAT guidelines for the diagnosis of IPF suggest that ANCA testing be considered only when systemic vasculitis is suspected on clinical grounds^8^; however, others have argued that detection of MPO‐ANCA antibodies through routine assessment at IPF/UIP diagnosis might enable early recognition.^6^, ^9^ Further uncertainties surround the management decisions and it remains unclear as to whether treatment should focus on immunosuppression and/or antifibrotic therapy. In our series, a number of additional patients with fibrosing ILD and positive MPO‐ANCA serology without evidence of systemic vasculitis remain under close observation. How best to manage these patients, especially given the adverse outcomes associated with IPF and immunosuppressive therapy^10^ remains unclear.

Fibro‐inflammatory tracheobronchial disease is a frequent manifestation of GPA^2^ and may result in life‐threatening airway complications. Similar to prevalence in existing literature,^11^ 17.1% of patients with GPA had tracheobronchial disease, manifesting as subglottic and main bronchial airway stenosis. As seen in our cohort, many patients require multiple therapeutic endobronchial interventions to alleviate symptomatic airway obstruction. The clinical dilemma often relates to whether the stenotic lesion occurs due to active inflammatory disease (and therefore immunosuppression responsive) or related to exaggerated post‐inflammatory cartilaginous fibrosis and stricturing. In our experience, resolving this uncertainty can be especially challenging in patients with ANV with isolated tracheobronchial involvement in whom a lack of surrogate markers of disease activity complicates decision‐making.

DAH is one of the most catastrophic manifestations of AAV. While induction therapy for AAV has greatly improved with the use of rituximab and/or cyclophosphamide in combination with high dose corticosteroids, the role of adjunctive TPE in DAH remains unclear.^12^ Observational data have been conflicting, and the recent prospective PEXIVAS trial failed to demonstrate conclusive benefit in this subgroup.^13^ However, PEXIVAS has been criticized for being underpowered for subgroup analysis and without pulmonary‐relevant outcome measures.^12^ Given the potential limitations of TPE, including the requirement for significant technical expertise, central venous catheterization and immunosuppression, the indications for TPE remain debated.^12^ In our practice, TPE was utilized as adjunctive treatment modality in select patients with more severe DAH, with the threshold also modulated by the presence and severity of concomitant acute kidney injury.

Consistent with published trial data,^14^ our practice has increasingly utilized rituximab for remission induction and maintenance; with all patients receiving rituximab‐based induction regimens since January 2018. Furthermore, following the publication of the PEXIVAS Trial in 2020,^13^ truncated corticosteroid tapering regimes have been successfully utilized, resulting in significantly lower steroid exposure, and associated morbidity.

While case series provide an important framework for understanding trends in the diagnosis and treatment of rare diseases such as vasculitis, future coordinated efforts to establish prospective clinical registries will be important for advancing vasculitis research in Australia and New Zealand.^3^

## AUTHOR CONTRIBUTIONS

Peter T. Bell and Gregory J. Keir designed the study. Peter T. Bell collected the raw data and wrote the first draft of the manuscript. Peter T. Bell, Robert Sheehy, Luke Droney, Kerri Prain, Richard Wong, and Gregory J. Keir edited the manuscript.

## CONFLICT OF INTEREST

None declared.

## ETHICS STATEMENT

This study was performed in compliance with the Declaration of Helsinki and according to approval granted by the local HREC (LNR/2021/QMS/74115).

## Data Availability

The data that support the findings of this study are available on request from the corresponding author. The data are not publicly available due to privacy or ethical restrictions.
